# On Animal and Vegetable Parasites of the Human Body; a Manual of Their Natural History, Diagnosis, and Treatment

**Published:** 1859-05

**Authors:** 


ART. V.—*On Animal and Vegetable Parasites of the Human Body; a Manual of their Natural History, Diagnosis, and Treatment.* By Dr. FREDERICH KUCHENMEISTER. Translated from the second German edition, by EDWIN LANKESTER, M.D., F.R.S. 2 vols., 8vo., pp. 738. Published by the Sydenham Society, London.

THE above is the title of a most elaborate treatise on the parasitic animals and plants of man. It is not merely a compendium of the labors of others, but, in addition, presents numerous and valuable observations of the author, Dr. Küchenmeister, one of the best of modern helminthologists.

It is now generally known that parasitic worms only reach maturity after passing through several stages of development, which occur succes-

---

sively in different animals, and sometimes exteriorly. Steenstrup, Küchenmeister, Von Siebold, Van Beneden, and others have carefully traced the successive development and migrations of some of the fluke and tape-worms, while the history of the thread-worms as yet remains in comparative obscurity.

As an example of the course of development of the tape-worms, attention may be directed to the history of the common tape-worm of man, (*Tænia solium,*) as traced by Dr. Küchenmeister.

1. The TAPE-WORM has a jointed, tape-like body, often many feet in length; and it inhabits the human small intestine. Its head is minute, and is provided with four circular suckers, and a short snout crowned with two circles of hooks.

2. The JOINTS, (*proglottides, or mature animals,*) which are constantly reproduced from the narrow portion of the tape-worm called the neck, are as constantly detached when perfected, and in this state are voided with the excrement. These separated joints are mature animals, with a hermaphroditic generative apparatus, and are self-impregnating. Under favorable circumstances, they may live and creep about for several days after expulsion from their host. The eggs they contain are more tenacious of life, and may be extruded or retained within the joints.

3. The EGGS, which may be swallowed by a hog, after extrusion, or with the parent joints, are hatched in the stomach of the latter animal.

4. The EMBRYOS thus produced, are minute globular sacs, provided with six hooks. They perforate the stomach of their host, and get into neighboring organs, or more frequently get into a blood-vessel, and are there whirled along with the current of blood until stopped in some distant organ: the liver, muscles, etc.

5. The embryo now loses its hooks, and becomes converted into a cyst about the size of pea, with a head like the parent tape-worm. This cyst is known as a HYDATID, or measle of pork, (*scolex, Cysticercus cellulosæ.*) By eating a piece of raw pork with such measles, the hydatids then become in the human small intestine the familiar tape-worm.

Dr. Küchenmeister describes four species of the tape-worm as found in man, of which one is a *Bothriocephalus,* and the others belong to the genus *Tænia.*

1. *Bothriocephalus latus,* as is well known, particularly infests the inhabitants of Russia and Switzerland. Its history has not yet been successfully traced. We have never seen this tape-worm from the inhabitants of the United States.

2. *Tænia solium* is the common tape-worm of Germany, England, and America. It is found associated with *Bothriocephalus latus* in France. In its larval condition it forms the measle of pork. The history of this species, carefully studied by Dr. Küchenmeister, has been given above as an instance of the mode of development generally of the tape-worms.

Dr. Küchenmeister remarks, vol. i. p. 114:—

"We find this *Tænia* very abundant wherever the breeding of pigs flourishes, as in Poland, Hungary, England, Pomerania, and Thuringia, and especially among those engaged in trades which bring them in contact with raw pork, and therefore with raw *Cysticerci*, (as butchers, cooks, eating-house keepers, etc.,) or in people who obtain portions of ready cooked or smoked sausages and hams from the butchers' shops. The most direct mode in which the transference may be effected, is, as experience has shown, the habit of eating raw pork. (Kleefeld's patient; Professor Merbach's observations; Thuringia, especially Nordhausen, Russia, Abyssinia.) Hence also, perhaps, arises the more frequent occurrence of *Tænia solium* in temperate zones, and especially in Central Europe.

"The importance of the subject may excuse the prolixity of the following remarks: The reason why we find *Tænia solium* so plentifully in butchers and their families, is, that the butchers contaminate their own hands in sausage-making, and also the blades of their knives in cutting up and selling meat. When they now wipe their mouths with the hands thus contaminated, or place the knife bedaubed with *Cysticerci* in their mouths, or, lastly, transfer these *Cysticerci* from the knife to the bread or sausages which they cut up for themselves, their families, or servants, the insignificant and scarcely perceptible *Cysticerci* are introduced into the mouth and swallowed. Cooks, and even housewives who cook and who have much to do with raw pork, infect themselves, partly by means of their hands or utensils, and partly by tasting the mixed masses of meat prepared from raw pork and other uncooked meats for the making of puddings before they are put into the frying-pan, or in the preparation of home-made sausages before drying.

"But we also see the possibility of the *Cysticerci* being introduced into private houses with the articles of animal food procured from the butchers' shops to be eaten without preparation, such as raw hams, and blood and liver sausages, especially when these articles of food are purchased by retail in small portions, when the *Cysticerci* have been transferred to them by the knife used in cutting them up. Thus, for example, my wife found *Cysticerci* in the water used in washing sausages, which must have been adhering to the hands of the butcher when filling the sausages, and thus have been transferred to the external surface of the gut. Lastly, the direct proof of these assertions has been furnished by myself in making the following experiments upon a murderer condemned to death: 72, 60, 36, 24, and 12 hours before execution, 12, 18, 15, 12, and 18 specimens of *Cysticercus cellulosæ* were administered to the criminal, partly in rice or vermicelli soup cooled to a blood heat, and partly in blood puddings from which the fat was removed and replaced by *Cysticerci*. The *Cysticerci* had already lain seventy-two hours in a cellar before I discovered them by chance, as I have stated further in 'Wittelshöfer's neuer medic. Wochenschrift, No. 1, 1855, and before I could administer them. The last administered had consequently lain about one hundred and thirty hours out of the living organism.

I hardly believe that those *Cysticerci* which had lain more than eighty hours were still capable of development, any more than I can believe this to be the case with the *Cysticerci* contained in smoked sausages and hams. On dissection, forty-eight hours after execution, I found ten young *Tæniæ*, of which indeed, six were deprived of their hooks, but four distinctly showed the hooks of *T. solium*. The little *Tæniæ* were 4–8 millim. in length, had exserted their hooks and proboscis, and attached themselves therewith to the intestine, and possessed a small band-like appendage, some 2–5 millim. long, which was notched or inverted at the extremity, as it is seen in those individuals which are found, for example, in the intestine of a dog three days after the administration of the *Cysticerci* of the rabbit."

We regard the *T. solium* as comparatively rare in the United States, which is rather remarkable when the fact is taken into consideration that hogs are extensively raised for food, though, as a counteracting influence to the production of tape-worms from the hydatids of measly pork, these are destroyed by the latter being used only after it is cooked.

*a. Cysticercus cellulosæ*, the scolex, larva, or hydatid of *T solium*, though having for its normal position the tissues of the hog, is occasionally found in similar parts of the body of man.

*b. Cysticercus ternuicollis*, another hydatid occasionally found in man, though belonging commonly to the domestic ox, sheep, horse, hog, etc. is the larva of the *Tænia marginata* of the dog.

We never met with these two species of *Cysticerci* in man in the United States. Our friend Dr. Weinland has recently described a new species, *C. acanthotrias*, obtained from a woman, at Richmond, Virginia.*

3. *Tænia mediocanellata* is a new species of tape-worm of man, discovered by Dr. Küchenmeister, in Germany.

4. *Tænia nana*, another new species, of small size, noticed by Dr. Küchenmeister, was discovered recently by Dr. Bilharz, in a boy in Egypt.

To these four tape-worms may now be added a fifth, recently described by Dr. Weinland, under the name of *T. flavopunctata*, and obtained from a child in Boston.

Dr. Küchenmeister describes two species of hydatid of the genus *Echinococcus* as parasitic in man; the two having been previously confounded together.

1. *E. scolicipariens* (*E. veterinorum* of earlier authors) occasionally found in man, is common in the domestic herbivorous animals. The experiments of Von Siebold and Küchenmeister, prove that it is transformed in the dog into a tape-worm, the *Tænia echinococcus* of Von Siebold.

2. *E. altricipariens* (*E. hominis, autorum*) is found in various organs of man and the domestic animals.

---

* An Essay on the Tape-worms of Man. By D. F. Weinland, Ph.D.; Cambridge, 1858.

Dr. Leidy met with this species, a few years since, thousands in number, contained in a cyst of about three inches in diameter, occupying the abdominal muscles, in the right iliac region, of an English sailor boy, in the dissecting rooms of the University of Pennsylvania. Though the subject had been injected with chloride of zinc, so as to bleach all the tissues of the body, three days subsequently the *Echinococci* were still living.

Besides the prophylaxis, the author gives a full account of the various modes of treatment for the expulsion of tape-worm.

Of fluke-worms, Dr. Küchenmeister describes five species as having been found in man. The two large species, *Distomum hepaticum* and *D. lanceolatum*, rarely found in man, are common in the biliary passages and portal vein of the deer, ox, sheep, etc.

*Distomum heterophyes*, Siebold, and *D. hæmatobium*, Bilharz, were both recently discovered in Egypt by Dr. Bilharz; the former in great numbers, in one instance, in the intestine of a boy; the latter very frequently in the portal system of veins.

The fifth species, *D. ophthalmobium*, Diesing, has only been found once, in Germany, in the eye of a child.

Of thread or round worms, Dr. Küchenmeister describes seven undoubtedly different species.

1. *Tricocephalus dispar* inhabits the neighborhood of the ilio-colic valve, that is to say, the terminal portion of the ilium and the commencement of the colon. *Trichina spiralis* of the human muscles is suspected to be the larva of the former.

Both the *Tricocephalus dispar* and *Trichina spiralis* have been a number of times observed in this country.

2. *Oxyuris vermicularis* infests the lower portion of the small intestine. The history of its successive stages of development is unknown.

According to our observations, this is the most common of all human intestinal worms; most individuals, perhaps, once in the course of a lifetime having acted the part of host to them in this country. We suspect that the larval form may be introduced into man, from the larvæ of insects, (in which they may pass a portion of their existence,) eaten in fruit, such as apples, pears, etc. We have found injections of ol. olivæ, ℥ij., ol. terebinth. ʒi., one of the most useful remedies in destroying this pest.

3. *Ascaris lumbricoides* is a common species in the human small intestine.

According to our observations, this is the next most common species of intestinal worm to the *Oxyuris vermicularis* in the United States; then follows the *Tricocephalus dispar*, with its suspected larval form, the *Trichina spiralis;* and lastly we have the *Tænia solium.*

4. *Strongylus gigas* has been occasionally found in the kidneys of man.

We have frequently observed it in the kidneys of the dog, wolf, and mink.

5. *Strongylus longevaginatus*, Diesing, (*Filaria bronchialis hominis*, Rudolphi,) has been observed a few times in the pulmonary tissue and bronchial glands.

6. *Ancylostomum duodenale*, Dubini, is a small nematoid worm, common in the human duodenum, in Egypt.

7. *Filaria medinensis;* the medina or guinea worm, one of the most serious in its effects of all human helminthes. It is common in tropical Africa and Asia, and was introduced into tropical America.

An immature *Filaria* has also been noticed in a few instances in the eye, in Germany.

Following Van Beneden and Vogt, Dr. Küchenmeister separates the genus *Linguatula* or *Pentastomum* from the true helminthes, and places it in the class of articulated animals, and with Vogt considers it as belonging to the order of Acarina (Mites.) Two species have been noticed in man.

1. *Linguatula constricta* is not uncommon, encysted in the livers of negroes in Africa. We would ask if it is not the same as *Pentastomum euryzonum*, Diesing, which Dr. Leidy and Van Beneden found in apes.

2. *Linguatula ferox*, or *Pentastomum denticulatum*, Rud., according to Zenker, as stated by Dr. Küchenmeister, is not uncommon in the liver of man, in Germany.

In a second family of the Acarina, Dr. Küchenmeister describes the common entozoon of the sebaceous follicles, the *Acarus folliculorum*.

Dr. P. H. Cabell,* of Richmond, Virginia, has described and figured a curious parasite found in company with the *Acarus folliculorum*. We do not know what to make of it. It is represented as having eight legs, like the *Acari*, but the femora and tibiæ are as long as those of insects.

In the family of the itch mites, *Acarida*, the author describes the common itch mite, *Acarus Scabiei*, and gives its history and treatment. The author further adds an account of the itch mites of the cat, dog, sheep, ox, and hare, as occasionally transferable to man.

In the family of the ticks, *Ixodida*, he describes the *Ixodes ricinus*, and the *I. marginatus*, and mentions three American species, the *I. americanus*, *I. humanus*, and *I. crenatus*.

The author also gives an account of the chicken louse, *Dermanyesus avium*, and the mites of cheese and dried fruits, as sometimes proving a source of irritation to man.

---

* Virginia Med. and Surg. Journal, vol. i. p. 14.

Of plant mites, Dr. Küchenmeister describes the *Leptus autumnalis*, as common in Europe, among gooseberry bushes and grass. During harvest it penetrates the skin of the reapers, often in immense numbers, producing troublesome itching, inflammation, and even fever. We have frequently observed a species of *Leptus* in similar positions in this country, but do not recollect of having heard of its being a source of annoyance to man.

Among the true insects, Dr. Küchenmeister gives a description, etc. of the human lice, of which he notices the *Pediculus capitis*, the *P. vestimenti*, and the *Phthirius pubis*. All of these have been observed upon the whites, blacks, and Indians of this country.

Of hemipterous insects, parasitic to man, the author describes the common bed-bug, *Acanthia lectularia*.

Among dipterous insects, Dr. Küchenmeister places the flea, of which he describes the two species; the common flea, *Pulex irritans*, and the chigger of tropical America, *Pulex penetrans*.

Under the head of true flies, the author refers to the larva of a species of bot-fly, or *Œstrus*, as parasitic in man, in South America. Thomas Say, a long time since, gave a description* of the larva of this *Œstrus*. Keferstein† has quite recently published a most elaborate paper, in which the question is discussed whether there is a true *Œstrus humanus*. The imago producing the *Œstrus* larva in man has not yet been positively ascertained, but it is suspected to be the *Cuterebra noxialis*, a bot-fly which is obnoxious to the cattle of South America. Dr. LeConte, of this city, informs us that the attack of the *Œstrus* on man is very common in Honduras. From some of his companions, who had the larvæ beneath the skin, about the shoulders, pectoral regions, and loins, he obtained a dozen specimens. The persons never knew when the eggs were deposited; nor was Dr. LeConte able to ascertain the character of the perfect insect.

Dr. Küchenmeister refers to the larvæ of the flower-flies *Anthomyia scalaris* and *A. canicularis*, and of the flesh-flies *Musca vomitoria*, *M. carnaria*, *M. domestica*, and *M. stabulans* as accidental parasites of man.

We have met with two instances in this city in which the larvæ of a species of *Anthomyia*‡ produced all the symptoms of ordinary cholera

---

* Jour. Acad. Nat. Sci., Phil., ii. 356.

† Verh. des Zool. Bot. Vereins in Wien, 1856, 637.

‡ In one instance the young larvæ appear to have been swallowed with some cold boiled cabbage. A number of individuals were expelled in both directions. Specimens still preserved present the following characters:—Larvæ from two to three lines long by one line broad, brown in color, semi-elliptical, divided into twelve segmorbus; and another instance in which pain and vomiting were produced by the larvæ* of the bluebottle-fly, *Musca vomitoria*?

As parasitic flies Dr. K. refers to the gnats *Tipulida*, and the sand-flies *Simulida*. To these we may add our mosquitoes, *Culicida*, and flies of the genera *Tabanus*, *Chrysops*, etc.

The second division of Dr. Küchenmeister's valuable work is devoted to the consideration of the vegetable parasites of man. He commences with the quotation: "All vegetable parasites which are found on animal bodies belong to the class *Cryptogamia*, and to the orders *Algæ* and *Fungi* exclusively."

Of Algæ, the yeast-plant, *Cryptococcus cerevisiæ*, is described first. It is found in yeast; and may be developed morbidly in the secretions of the alimentary canal.

The *Merismopœdia*, or *Sarcina ventriculi*, is described as a not unfrequent algous parasite of the human stomach, and is mostly met with through vomiting. The author observes that neither Virchow nor Lebert ever saw this plant associated with the preceding. We saw the two plants abundantly commingled in a frothy liquid, vomited by an adult male patient in St. George's Hospital, in London, in June, 1850.

*Leptothrix buccalis* is the remarkable filamentary plant so constantly found in every one's mouth.

Dr. Küchenmeister describes six species of *Leptomitus*, of which *L. urophilus* is found in unhealthy urine; *L. Hannoverii*, found on the tongue and pharynx in typhus, pneumonia, pleurisy, etc.; *L. epidermidis*, found once in eczema-like vesicles of the epidermis; *L. uteri* and *L. muci uterini* found in uterine discharges; and *L. oculi*, found in the eye.

Lastly, the author describes as an alga a filamentary plant found in the intestines, the *Oscillaria intestini*.

All the algæ, except the *Leptomitus oculi*, appear to be innocuous.

Of fungi, Dr. Küchenmeister describes fourteen species which have been found parasitic in man. Many of these not only accompany, but appear to be

---

ments, which are minutely shagreened, furnished dorso-laterally each with six posteriorly divergent spines, and ventrally with a transverse row of small conical tubercles; head bipapillate, with a pair of hooks; caudal segment obliquely truncated, provided with two elevated spiracles, and fringed with six spines.

* These larvæ are six lines long and one line broad, anteriorly acute, posteriorly obtuse, minutely shagreened; and the segments each provided with a transverse row of minute tubercles; head bipapillate, with a pair of hooks; caudal segment complex: dorsally having a large pit margined with a coronet of tubercles, and furnished at bottom with a pair of spiracles; ventrally on each side of the anal aperture there is a triangular, brown tubercle, and behind it, a transverse ridge terminating in a pair of conical tubercles.

the cause of troublesome cutaneous diseases, which are contagious through the agency of the fungous spores.

The *Trichophyton tonsurans* is the cause of Porrigo scutulata or herpes tonsurans.

A second species of *Trichophyton* accompanies Plica polonica; and a third species was detected by Lebert in the scabs of an ulcer of the leg.

*Microsporon Audouini* accompanies Porrigo decalvans; a second species, *M. mentagrophytes*, is the cause of Mentagra; and a third, *M. furfur*, is the cause of Pityriasis versicolor.

*Achorion Schœnleinii* is the cause of Favus.

*Oidium albicans* is the fungus plant of Thrush or Aphthæ.

The remaining fungi are as follow: One of the lungs, several times noticed; two of the external auditory meatus; the nail fungus; *Mucor mucedo* from a cavity of the lungs; and *Puccinia Favi* accompanying *Achorion Schœnleinii* in Favus.

In conclusion, we take the opportunity of recommending the highly valuable work of Dr. Küchenmeister to the attention of American physicians, and hope for their benefit it may be republished in this country.

